# A verification strategy for web services composition using enhanced stacked automata model

**DOI:** 10.1186/s40064-015-0805-1

**Published:** 2015-02-27

**Authors:** Danapaquiame Nagamouttou, Ilavarasan Egambaram, Muthumanickam Krishnan, Poonkuzhali Narasingam

**Affiliations:** Department of Computer Science and Engineering, Pondicherry Engineering College, Pondicherry, 605014 India

**Keywords:** Web service composition, ESAM, Promela, BPEL4WS, SPIN

## Abstract

Currently, Service-Oriented Architecture (SOA) is becoming the most popular software architecture of contemporary enterprise applications, and one crucial technique of its implementation is web services. Individual service offered by some service providers may symbolize limited business functionality; however, by composing individual services from different service providers, a composite service describing the intact business process of an enterprise can be made. Many new standards have been defined to decipher web service composition problem namely Business Process Execution Language (BPEL). BPEL provides an initial work for forming an Extended Markup Language (XML) specification language for defining and implementing business practice workflows for web services. The problems with most realistic approaches to service composition are the verification of composed web services. It has to depend on formal verification method to ensure the correctness of composed services. A few research works has been carried out in the literature survey for verification of web services for deterministic system. Moreover the existing models did not address the verification properties like dead transition, deadlock, reachability and safetyness. In this paper, a new model to verify the composed web services using Enhanced Stacked Automata Model (ESAM) has been proposed. The correctness properties of the non-deterministic system have been evaluated based on the properties like dead transition, deadlock, safetyness, liveness and reachability.

Initially web services are composed using Business Process Execution Language for Web Service (BPEL4WS) and it is converted into ESAM (combination of Muller Automata (MA) and Push Down Automata (PDA)) and it is transformed into Promela language, an input language for Simple ProMeLa Interpreter (SPIN) tool. The model is verified using SPIN tool and the results revealed better recital in terms of finding dead transition and deadlock in contrast to the existing models.

## 1 Introduction

**Figure 1:**
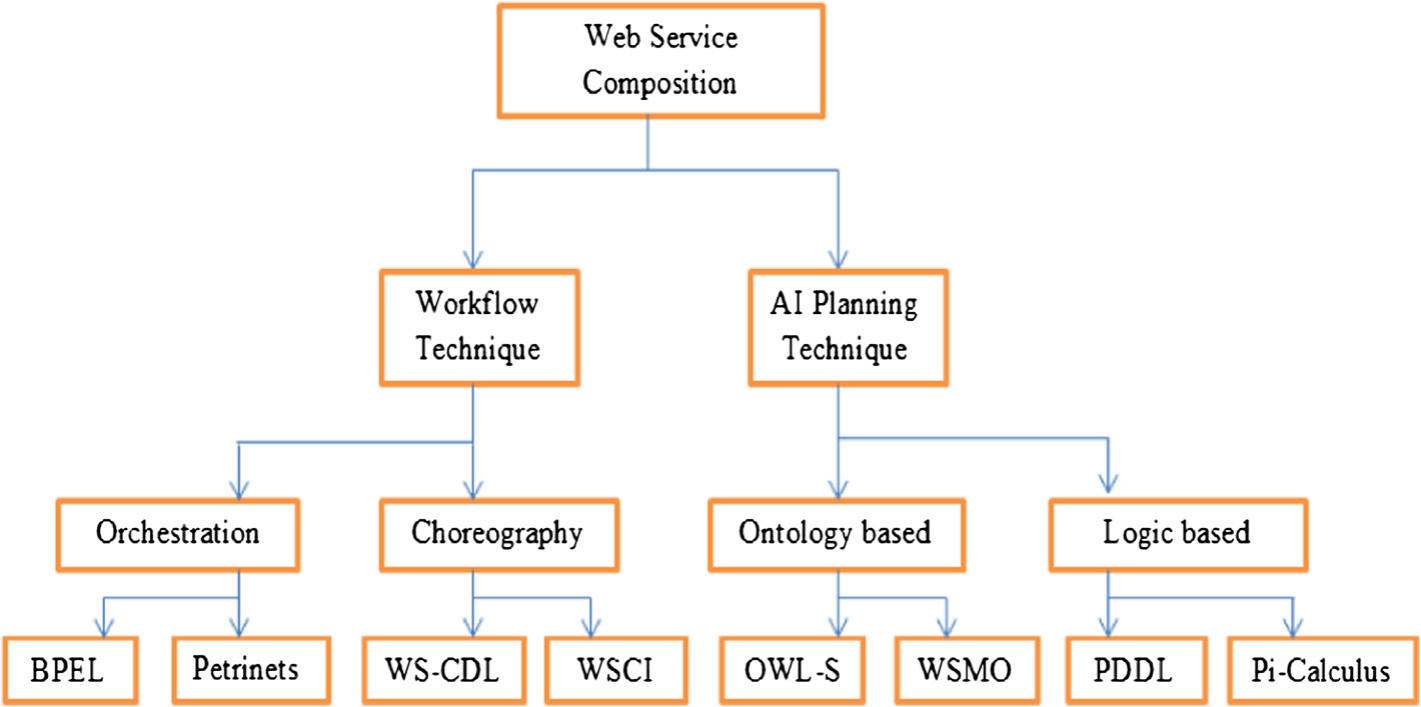
**Flow of web services composition.**

Web services are disseminated and self-sufficient computational elements that solve specific tasks, varying from undemanding requests to multifaceted business processes and the information will be interacted using XML messages following the SOAP standard. Composition of services thus received much interest to support B2B. The business world has developed a number of XML-based standards to formalize the specification of web services, their composition, and their execution. Web service composition should satisfy several fundamental requirements: Connectivity, support for non-functional quality of service metrics, correctness, scalability, and in the desiderative situation. Web services composition can be broadly classified into two techniques namely Workflow and AI Planning as shown in Figure 1. Workflow technique can be further classified into orchestration and choreography. In orchestration, the involved web services are under control of a single endpoint central process (another web service). Choreography, in contrast, does not depend on a central orchestrator. Each web service that participates in the choreography has to know exactly when to become active and with whom to interoperate. Choreography is based on collaboration and is mainly used to exchange messages in public business processes. BPEL is one of the types of Orchestration and is used for composing the web services. Web Service Choreography Description Language (WS-CDL) and Web Service Choreography Interface (WSCI) comes under choreography. Certain applications use WS-CDL and WSCI for choreography. Ontology Web Language for Service (OWL-S) and WSMO come under ontology based language. PDDL and Pi-Calculus comes under Logic based approach. Even with the availability of many languages, BPEL is preferred for web service composition in the real time applications.

Web services are composed using BPEL4WS and it is transformed into ESAM (grouping of Amend Muller Automata and Push down automata) and it is changed into Promela language, an input language for Simple ProMeLa Interpreter (SPIN) tool. The model is verified using SPIN tool. Only a few cases considered the verification part, deterministic systems in particular. This paper intends to compose web services and verification of web services for non-deterministic systems with the avoidance of dead transition and deadlock.

**Figure 2:**
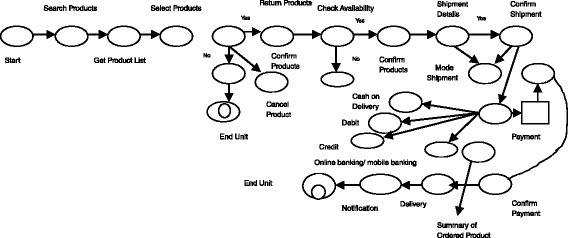
**Deterministic finite automata.**

**Figure 3:**
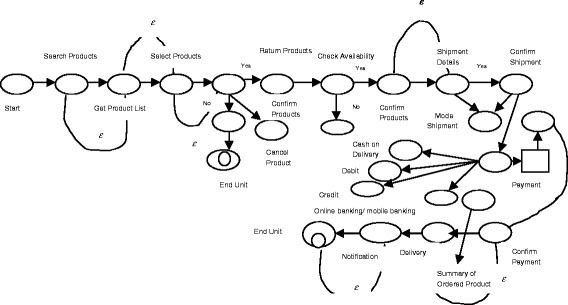
**Non-deterministic finite automata.**
- The proposed model is also compared with the Timed Automata (Su et al. 2009), Interface Automata (Cambronero et al. 2011) and ColoredPetrinet (Yang et al. 2005).
- Finally, SPIN tool is used for verifying the composed web Services (input is thePromela)
- The report shows that our system is giving more efficient result than the other system in terms of finding Dead transition and deadlock.
- Our system is readily applicable for any existing applications, and it could be efficiently used for verifying composed web services.

The main contribution of this paper to be verification of the composed web services which is summarized as follows:

- Here a new model to verify the composed web services using ESAM was proposed. ESAM is the combination, of Amend Muller Automata and Push down automata which is suitable for both deterministic and non-deterministic system. Deterministic system means, on receiving input it goes to one state only as shown in Figure 2, whereas non-deterministic system, on receiving same input it goes to many states as shown in Figure 3.

The rest of this paper is organized as follows. Section 2, describes related works. Section 3, describes background, application overview and Automata definition. Section 4 describes proposed Architecture. Section 5, depicts the algorithm for composition. Section 6, depicts the verification algorithm. Section 7, portrays the sample flow of airline reservation system. Sections 8 discuss the implementation of proposed model for web service composition and verification. The performance of the proposed model is evaluated and reported in this section. Section 9, describes the conclusion and give an outlook on future research.

## 2 Related works

The development of composite web services is still an emerging technique; there is a need for effective and efficient composed web services by using formal technique. We now discuss related work on web service composition verification. The composition and verification of web services are generally classified into four categories. They are, timed automata, interface automata, colored Petrinet and model checking.

Timed Automata were used for verification of composed web services (Cambronero et al. 2011; Jin Song et al. 2006). Web service choreography description language (WSCDL) was used for composition of web services, converted into Timed Automata and verified using the tool called UPPAAL. Here, only emptiness problem is verified. The whole process is only in the design level and not in the implementation level. Timed Automata applicable only for deterministic system rather than non-deterministic system.

Interface automata were used for verification of composed web services (Su et al. 2009; Alfaro & Henzinger 2001; Holzmann 1997). Initially composition of web services are done by BPEL4WS and converted into Interface automata. This IA is mapped into Promela. Finally it is verified using the tool SPIN. This approach is applicable for deterministic system rather than non-deterministic system.

Petrinet model were used for verification and validation of composite web services (Lohmann 2007; Foster et al. 2006; Hamadi and Benatallah 2003; Verbeek & van der Aalst 2005; Yang et al. 2005; Schmidt & Stahl 2004; Yi & Kochut 2004; Narayanan & McIlraith 2002). It presents a methodology for the design, verification and validation of composite Web Services using WS-CDL as the language for describing Web Service interactions and Petrinet as a formalism (that allows us to analyze the described systems). They have considered timed automata and prioritized collaboration in composite Web Services, so the considered model of Petri nets is a prioritized version of Timed Petri nets.

Model checking is used for webs service composition (Foster et al. 2003a; Huang & Mason 2006). Specifications of the design are modeled in UML in the form MSSC and compiled in FSP. Verification process applied for the following steps to model, specify properties of the composition, and implement the example in BPEL4WS.

1. Translate BPEL4WS - > FSP (used LTSA-MSC)
2. BPEL4WS implementation
3. Generate BPEL4WS model in FSP for use within LTSA

Used LTSA model checker to detect possible additional scenarios, checking and iterate tracing resolution until no violations or deadlocks are discovered depends on the model designed. It is suitable for deterministic system rather than non- deterministic system.

Foster (Foster et al. 2003b), defined a model based on analysis of obligation in web service choreography. This paper proposed composition of web services and implementation of WS-CDL at design level. The verification part was not addressed.

Alves et al. 2007; Xiwu & Zhengding 2006 suggested about the BPEL language representation for the business processes. This language is used for composition of web services and verified using automata.

Samira (Samira et al. 2008) suggested web service interactions using coordination language Reo. This paper proposed an approach to derive the formal semantics of WS-BPEL processes compositionally using Reo and constraint automata. Mapping each will result the behaviour of process. They did only composition of web services by using Reo and constraint automata and not the verification part.

In (Jin Song et al. 2006), Jin Song Dong contributed the concept of threefold, described as

1. Defined automata based semantics for the Orc language, which allows a systematic constriction of timed automata models from Orc models.
2. They explored ways of use Uppaal to verify critical properties over Orc models.
3. Developed a toll to automate our approach.

Here, it uses the semantics verification using the tool called Uppaal and Orc does not support the complex data structure. So a new approach should be used for manipulating those complex date structure, it is applicable for deterministic system rather than non-deterministic system.

Fu,x. (Fu et al. 2004) suggested that, BPEL specifications of Web Services are translated to an intermediate representation (Guarded Automata) with unbounded queries for incoming messages. Guarded Automata can then be converted into Promela language and it is verified using the tool called SPIN. Guarded Automata is applicable for deterministic cases rather than non-determinism.

Co, k-m.et (Kuo-Ming et al. 2002) described the analysis of grid service composition with BPEL4WS. They have used BPEL4WSas a business workflow description language for the composition of grid services. It proposed a high-level architecture to compliment GSI + BPEL4WS for defining process workflow among grid services. During composition, depending on the specification of OGSI, it is mapped with BPEL4WS for defining the process workflow. They have used only composition and not verification.

In (Hou et al.), HovLisn, ontology based approach for verification of web services composition is discussed. Web service interactions with pi- calculus, an automatic mechanism were used and converting conceptual capability description to formal process.

The Table 1 shows that the overall survey of my research works. Here only limited number of models is available for verifying the composed Web Services. Even though number of the models is available, it is not satisfying the properties like reachability, dead transition, deadlock and safetyness.

**Table 1:** **Comparison of existing work with proposed work**

**S. No**	**Researchers**	**Formal model**	**Transformation verification**				**Type of system**	**Specification**	**Tool used**	**Level of development**
			**Correctness property**	**Safetyness**	**Dead transition**	**Deadlock**				
1	M.Emilia Cambronero	Timed Automata	No	No	No	No	Deterministic System	WSCDL	WST and UPPAAL	Design Level
2	Jia Mei	Interface Automata	No	No	No	No	Deterministic System	BPEL, Promela	SPIN	Implementation Level
3	Jin Song Dong	Orchestration on computation via Timed automata	No	No	No	No	Deterministic System	LTL	UPPAAL	Design Level
4	Valentin Valero	Colored Petrinet Method	Yes	Partial	Partial	No	Deterministic System	BPEL	CPN	Implementation Level
5	Guangquan Zhang	Refinement Checking Method	Yes	No	No	No	Deterministic System	BPEL	UPPAAL	Design Level
6	Our Work	Modified Muller Automata	Yes	Yes	Yes	Yes	Both Deterministic and non-Deterministic System	WSCDL	-	Implementation Level

Earlier studies achieved web service composition verification for deterministic systems. The properties of dead transition and deadlock were not handled. Our paper captivated to readdress these issues in non-deterministic system during verification of composed web services.

## 3 Background

An automaton is a mathematical model that represents the behavior of the systems with the help of discrete number of inputs and discrete number of outputs. Deterministic system is defined as on receiving input, it goes to one state.

Figure 2 shows the example of deterministic finite automata .it contains six states namely Q_0_, Q_1_, Q_2_, Q_3,_ Q_4_ and Q_5_. Giving input “a” to Q_0_ it goes to Q_1_state only. Double circle is represented as final state. On receiving an input “a”, it does not go to more than one state.

The Figure 3 shows the transition diagram for the non - deterministic System. It contains six states namely Q_0_, Q_1_, Q_2_, Q_3,_ Q_4_ and Q_5._ Giving an input a to Q_0_ it goes to Q_1,_ Q_3_ and Q_4_states. Double circle is represented as final state.

Deterministic system avoids the problem like reachability and emptiness to an extend of 40 to 50%, suitable for Timed automata and Interface Automata. Whereas non-deterministic System avoids the problems like Reachability, Emptiness, Dead Transition, and Deadlock at 95 to 99%.

### 3.1 Application overview

A Net Beans module is a set of Java classes written to interact with the Net Beans APIs, for extending the IDE or for creating your own application on the Platform.

JAX-RPC is used for creating web services that can be supported in the IDE. Using the JAX-RPC web services, system gets the “JAX-RPC Web Services” plug-in from the Plug-in manager.

The Business Process Execution Language for Web Services (BPEL4WS), also referred to as BPEL, is currently a de facto standard for building, specifying and executing business processes for web services composition and orchestration. BPEL composes web services to get a specific result. The composition result is named a *process*, involved services are called as *partners*, and the message exchange is referred to as an *activity*. In other words, a process contains a set of activities and it invokes external partner services using a WSDL interface.

A BPEL process defines the order in which involved web services are composed, either in sequence or in parallel. BPEL allows describing conditional activities. An invocation of a Web service can for example rely on the result of another web service’s invocation. With BPEL, it is possible to create loops, declare variables, copy and assign values as well as to use fault handlers. Complex business processes can be built algorithmically by using all these constructs. It can be helpful to describe business processes graphically through UML (Unified Modelling Language) activity diagrams.

BPEL supports two different ways of describing business processes that support orchestration and choreography:

1. Executable processes allow for specifying the details of business processes. They follow the orchestration paradigm and can be executed by an orchestration engine.
2. Abstract business protocols allow specification of the public message exchange between parties only. They do not include the internal details of process flows and are not executable. They follow the choreography paradigm.

### 3.2 Web service orchestration basics

The IDE's BPEL Designer provides a highly graphical environment for authoring, deploying and testing web-service centric business processes. This is often called web service orchestration and is one of the keystones of service-oriented architecture (SOA). A BPEL process can be thought of as a logical aggregator and coordinator of web services. In such a process, a collection of partner web-service components can collaborate synchronously or asynchronously, participate in long-lived conversations, and support fault handling. Thus, the IDE's BPEL Designer feature extends the power of service-oriented architecture.

The BPEL modeling environment includes deployment runtime, and the ability to author, edit, test-run, and debug BPEL processes. The BPEL Designer feature lets you use drag-and-drop functionality to create visual diagrams of business processes to orchestrate web services. The BPEL Designer feature supports two-way round-trip engineering of processes that are expressed in the Web Services Business Process Execution Language Version 2.0 (WS-BPEL 2.0, or generically, BPEL). In the BPEL Designer, you can create a business diagram in the visual Design view or manipulate source code in the Source view. The BPEL source code and its visual diagram are always kept in synchronize.

#### Definition 1 (Amend Muller Automata)

AMA is a type of a ω-automaton (ω-automata are finite automata on infinite words). The Amend Muller automata is defined using Muller acceptance condition, i.e. the set of all states visited infinitely often must be an element of the acceptance set. An AMA is a tuple *A* = (*Q*_*ama*_*,* Σ_*ama*_*,* δ_*ama*_*, Ѓ*_*ama,*_*q*_*0aa*_*, F*_*ama*_) that consists of the following components:

- *Q*_*ama*_ is a finite set. The elements of Q are called the states of A
- Σ_*ama*_ is a finite set called the alphabet of A
- δ_*ama*_: *Q*_*ama*_*×* Σ_*ama*_ → *Q*_*ama*_ is a function, called the transition function of A.
- *q*_*0ama*_ is an element of *Q*_*ama*_*,* called the initial state.
- *Ѓ*_*ama*_ is an stack symbol contains the information about the state regarding the operations like Push and Pop.
- *F*_*ama*_*⊆ Q*_*ama*_ is the acceptance condition. A accepts exactly those runs in which at least one of the infinitely often occurring states is in F_*a*ma_.
- M accept a ω-word α∈∑^ω^ if and only if there exist a run r of M on α satisfying Inf(r) εF_m_ i.e. the set of infinitely often visited states are exactly one of the set in F_m_.

#### Definition 2 (Push down Automata)

- A push down automata M is defined by (*Q*_*pda*_*,* Σ_*pda*_*, Ѓ*_*pda*_*,* δ_*pda*_*, q0*_*pda*_*, z0*_*pda*_*, F*_*pda*_) Where,
- *Q*_*pda*_ is the finite set of states
- Σ_*pda*_ is the alphabet called input alphabet
- *Ѓ*_*pda*_ is the stack alphabet, is a finite alphabet of stack symbols.
- *q*_*0pda*_ ε *Q*_*pda*_ is the stack state/initial state
- *z*_*0pda*_ in *Ѓ*_*pda*_ is the particular stack symbol called start symbol.
- *F*_*pda*_*≤ Q*_*pda*_ is the set of final states.
- δ_*pda*_ is the transition relation.

### 3.3 Promela and spin

PROMELA is the language used in SPIN to represent concurrent systems with abstraction. PROMELA programs consist of processes, message channels, and variables. Processes are global objects that represent the concurrent entities of the distributed system. Message channels and variables can be declared either globally or locally in a process. PROMELA supports rendezvous and asynchronous communication between processes via channels. Processes specify behavior, while channels and global variables define the environment in which the process runs.

SPIN is a verification tool for composed web services. SPIN takes a model of the system design with a requirement as input and the model checking algorithm specifies whether the system design meets the requirement or not. SPIN verification is focused on proving the correctness of process interactions; not much importance is given to internal computations of the processes.

In the Web service composition part the related services are invoked and composed using BPEL4WS. While composing, the partner links between the services are identified. Based on the established links the composition process has been completed. The Web services are created by using Java through Net Beans. After completing the composition, the BPEL file is mapped into Muller automata notations and saved as xml files. For retrieving the xml content from xml file “XPath” xml query language is used. Services are retrieved from xml for converting into Promela and it is stored as variable in pml file and if service is available, it gets increment or it returns service is zero. The xml file is transformed into .pml file and pml file is given as input to spin tool for verifying the composed services. Spin tool will generate the automata diagram for the given pml input and safety, Reachability property is verified using that tool. An algorithm is designed for verifying the dead transitions present in the diagram.

## 4 Proposed architecture

**Figure 4:**
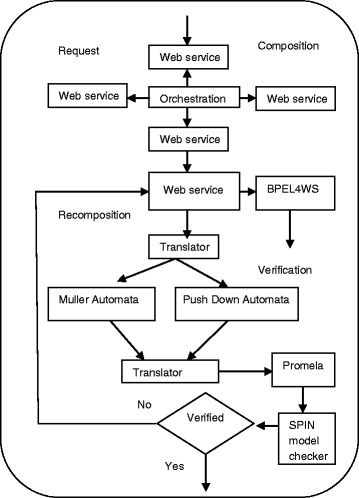
**Proposed architecture.**

Our proposed architecture has two parts is shown in Figure 4. First one is Web services composition and second is verification of composite Web services. In the composition part, the user request is collected, which is sent to central controller and the central controller will be interacting with neighbour web service request. Based on the request the related Web services will be invoked. The invoked services are composed by using BPEL4WS.The output of the composition part will be given as the input to the verification part. During verification the BPEL4WS is mapped into Amend Muller automata and Push down automata with the help of translator. After mapping, it is transformed into Promela and it is input to the SPIN Model checker. Finally, it is verified using SPIN tool for checking the correctness properties of web services composition.

Orchestration is evidently more proficient and stretchy when it comes to composed web services to execute business processes. It has the following advantages over choreography:

- The coordination of process components is centrally managed by a specific coordinator. Web services can be incorporated without being aware that they are taking part in a larger business process.
- If any faults occur during verification, it has two options to satisfy the request. Either modify the original service or select alternate service depends on the user satisfaction.

## 5 Algorithm for composition based on automata

The proposed system concentrates on composition and verification of web services composition. The algorithm for the web services composition is shown below.

The semantic description of the composition of web services is illustrated in the following definition.

### Definition 3

A composition schema is a tuple (S, M) where S = (s_1_, s_2_…, s_n_) is the set of services, M is the set of messages. Each service S_i_ = {M_i_^in^, _Mi_^iout^} is a pair of disjoint set messages (M_i_^in^∩M_i_^out^ = ϕ) where

- M_i_^in^ = set of incoming messages
- M_i_^out^ = set of outgoing messages and
- M_i_ = M_i_^in^ U M_i_^out^ set of messages of services where
- U_i_ε [1…n] M_i_^in^ = U_iε [1…n]_ M_i_^out^ = M

We assume that each message s a unique sender and a unique receiver and service cannot send a message back to itself.

For example

- Services are S = {Transport, Airline, Hotel}
- Set of Messages = {query, suggest, confirm, reserve}
- The input and output messages of service types are defined as M_agent_^in = {query}^
- ^M^_agent_^out^ = {suggest}
- ^M^_smith_^in^ = {suggest, confirm}
- ^M^_smith_^out^ = {query, reserve}
- M _hotel_^in^ = {reserve}
- M_hotel_^out^ = {confirm}

### Definition 4

Let X = {S, M} be a composition schema. The set of services over X is a tuple R = ((S, M), A) where

A = Finite state automaton with Amend Muller automaton alphabet M. We let L(R) = L(A). I.e. the language recognized by A.

Muller automata constructs the set of states (is transitions) {q_0_, q_1_, q_2_, q_3_, q_4_, q_5_………q_n_).

The initial state = {q_0_}

The final state = {q_n_}

The alphabet = {(query, suggest, confirm, reserve)}

The set of transitions = {(q_0_, query, q_1_)}, (q_1_, suggest, q_2_), (q_2_, query, q_3_), (q_3_, suggest, q_2_), (q_2_, reserve, q_4_), (q_4_, confirm, q_5_).

The regular expression for the above is defined as

Query suggest (query suggest)*reserve confirm

## 6 Algorithm for verification

The algorithm is designed for the checking dead transitions in the automata diagram. Dead Transitions is stated as “the transitions which will never be enabled”. There are no activities in the process that cannot be realized. If initially dead transitions exist, then the composition process is bad designed.

### 6.1 Amend Stacked Muller Algorithm for avoiding dead transitions in services

The algorithm initially create service matrix and found the length of the states. It sets the matrix value and it sets 0 if the initial and final states are equal, otherwise sets as 1. Then it find the position of the given accepted state and if the count value is 0 or greater than length of the states means it sets the Boolean variable “dead” as true. Otherwise, it checks if the given accepted state is the initial state and set “dead” as true else set “dead” as false.

### 6.2 Service composition verification

During verification process the following properties are verified. They are

**Safety** assurance that the composition is deadlock free and is checked against partial correctness of transitions

**Correctness** assurance that the composed services are correct

**Reachability** assurance that whether it is possible for a process to achieve the desired result.

**Liveness** assurances against starvation of progress (that the service process eventually terminates) and that messages received are served on a first-come-first-served basis.

**Dead transitions** means that the transitions which will never be enabled. There are no activities in the process that cannot be realized. If initially dead transition exists, then the composition was bad designed.

**Deadlock** is a situation in which two or more competing behaviour are each waiting for the other to finish, and thus neither ever does

## 7 Sample flow for airline reservation system

**Figure 5:**
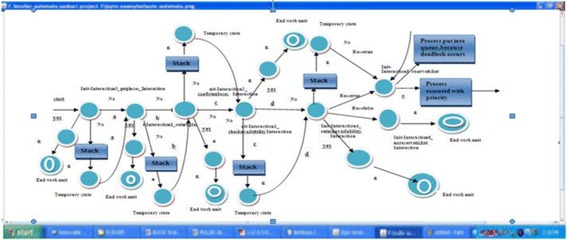
**Automata diagram for airline reservation system.**

The diagram in Figure 5 shows the flow of reserve ticket for Airline Reservation System. The process is started by Init_Interaction0_getplaces_Interaction. Giving input “a” on this state it goes to Init_Interaction1_returnplaces_Interaction. Giving input “b” on this state, it goes to Init_Interactio2_confirmplaces_Interaction. Similarly up to “e” input it reaches to the desired state. On Init_Interaction_Returnavailability_Interaction state, traffic occurs. The transition is redirected to stack and to temporary state with help of “ε”. Finally it is reached to the destination with condition satisfied (Res = true). All the states maintaining stack i.e. push down automata. If any traffic or deadlock occurs, the transition is redirected to stack. Fetching the information from stack and it is passed to temporary state. Stack contains the top of the symbol is “ε”. So it is easily transited to another state with the help of “ε”. If the condition is not satisfied it goes to end work unit.

## 8 Case study

The significance of Web Services for electronic government has become more and more increasing, because of providing services and exchanging information related to the government management.

In this Case study, the applications are Ticket Reservation, Hotel Reservation and Transport Reservation and these services are composed using BPEL4WS by creating a BPEL module. In this module, BPEL process diagram will be generated by invoking the basic and structured activities of BPEL and we need tocreate a new composite Application project. Finally, add a JBI Module project to the composite application project. Test-run a business process is to be performed, make sure that the application Server is started and build the BPEL module project. Add it as a JBI Module to our Composite Application project and deploying a Composite Application project to the JBI server. The Deploy action compiles the files in the Composite Application project, packages the compiled BPEL and related web service artifacts (.wsdl and .xsd files), and deploys them to the BPEL Service Engine.

After composition, the verification part is started by mapping the BPEL files to ESAM notations as xml files. For converting into Promela, services are retrieved from xml using “XPath” and it is stored as variable in pml file. The XML Path Language known as XPath, is a query language for selecting nodes from an XML document. The XPath language is based on a tree representation of the XML document, and provides the ability to navigate around the tree, selecting nodes by a variety of criteria. The xml file is transformed into .pml file and pml file is given as input to spin tool for verifying the composed services. Spin tool will generate the automata diagram for the given pml input and verify the properties like deadlock, dead transition, safety, and reachability.

Hotel reservation lists out the availability of hotel in a given district. Also, it provides the services for checking the room availability and reserving the room, etc. Transport service includes the operations like get transport availability, check availability and reserve transport, etc. Ticket Reservation includes the operations like get places, returnplaces, confirm places, ticket availability, reserve ticket, etc.

### 8.1 Simulation of automata

The spinspider tab is opened and automata is choosed for generating the automata diagram.

**Figure 6:**
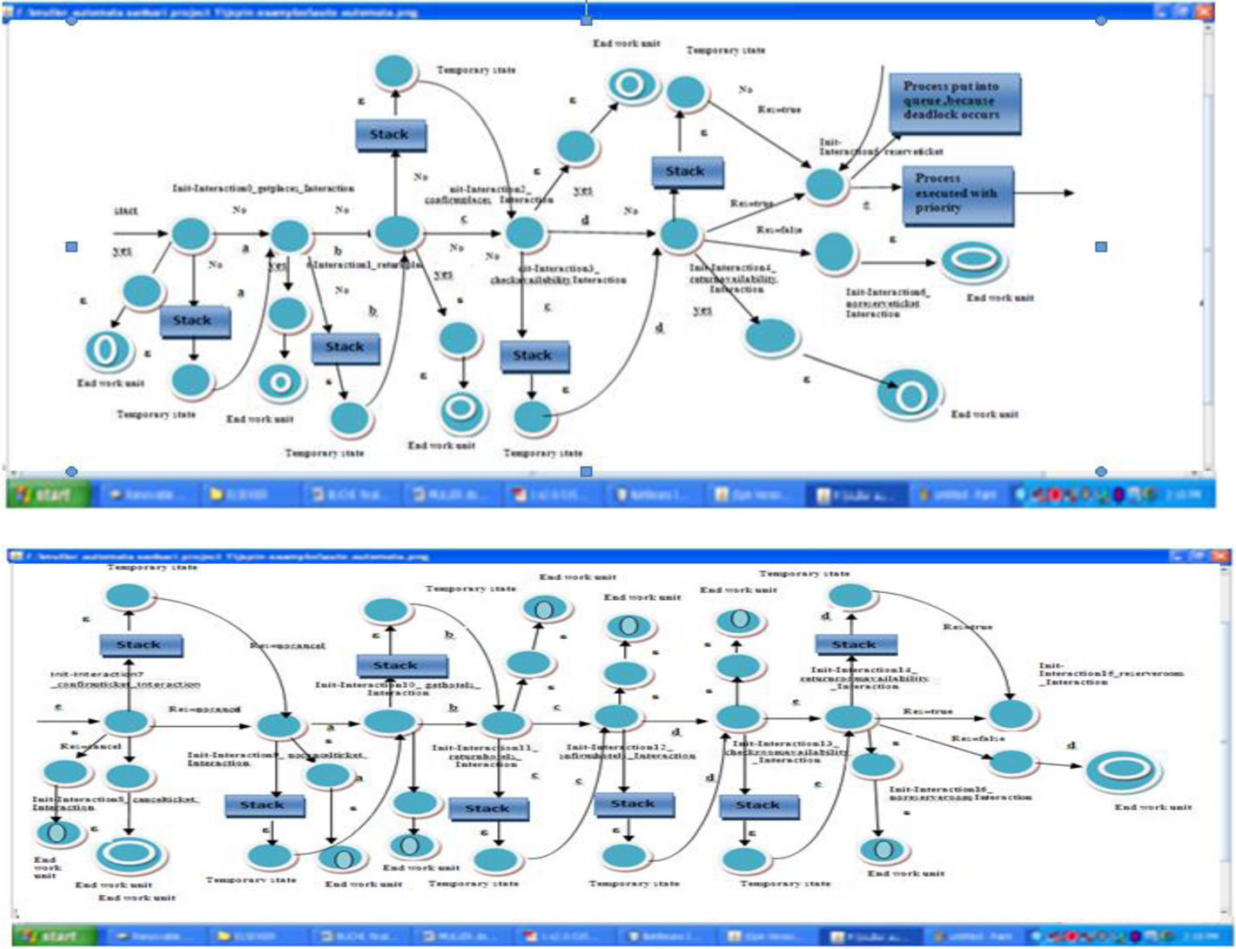
**Transition diagram for airline reservation system.**

The diagram in Figure 6 shows the flow of reserve ticket for Airline Reservation Systemusing SPIN tool. The process is started by Init_Interaction0_getplaces_Interaction. Giving input “a” on this state it goes to Init_Interaction1_returnplaces_Interaction. Giving input “b” on this state, it goes to Init_Interactio2_confirmplaces_Interaction. Similarly up to “e” input it reaches to the desired state. On Init_Interaction_returnavailability_Interaction state, traffic occurs. The transition is redirected to stack and to temporary state with help of ”ε”. Finally it is reached to the destination with condition satisfied (Res = true). All the states maintaining stack i.e. push down automata. If any traffic or deadlock occurs, the transition is redirected to stack. Fetching the information from stack and passed to temporary state. Stack contains the top of the symbol is ε. So it is easily transited to another state with the help of ε. If the condition is not satisfied it goes to end work unit.

Here dead transition algorithm is verified. If no transition occurs on the particular state, there will be problem in the composition and hence recomposition will be done. If any deadlock occurs, it is redirected to stack with the help of empty string as input. Here deadlock property is verified. Consider a case where room is reserved by some person, transport is reserved some other person and at the same time a third person asking for both the reserved room and transport. Now he has to wait until the resources used by the existing person must be released, which leads to deadlock. This can be avoided by using preemption which means allocating priority to the processes. Depends on this, if any higher priority process enters, the lower priority process will preempt its task in to the queue, higher priority process will be executed. After finishing its task, waiting process will be resumed. Following algorithm shows the deadlock evasion.

### 8.2 Amend Stacked Muller Algorithm for deadlock evasion in composed web services

The following algorithm shows the evasion of the deadlock for the composed web services. If the state (services) has resources R and state has equal and higher prioritythan resources, it will refuse the request and if the resource does not exists for the particular state then also it will refuse the request. Otherwise if the resource is not free which is used by some other services, it will be stored into the queue. If the stored services are at the front of queue, it will grant services exclusive access to resource R. Like this way deadlock evasion is carried out.

### 8.3 Performance measurements

1. Specification of data set

Performance measurements were based on a data set of workflows in the Figure 6. The circle labeled with services Sama (Start Amend Muller automata) represents the start state, Fama(Final Amend Muller Automata) represents final state, Transition represents the flows from one state to another state.

2 Results

Response time for the different models s been calculated. Response time is the difference between submission of starting time and total time taken to finish the task.

Response Time = Starting time for Transition – Total time taken to finish the task.

RT_ESAM_ = ST_ESAM–_TA_ESAM_

Here the response time for CWS-PNs methodology is higher than proposed model and the Timed automata model. Response time for timed automata model is less, because, it set the clock for each state, if time elapsed, the transition failed or if the transition does not exists due to deadlock or some situation, it fails to reach the desired state. When comparing timed automata model and proposed model, proposed model gives high response time than timed automata. Even though the response time is higher in the proposed model, it does not meet the deadlock or dead transition. Table 2 shows the Response time calculation for different models like ESAM, timed automat and colored petrinet. ESAM is taken less time than TA and CPN.

**Table 2:** **Response time calculation for different models**

**States**	**Proposed model-Amend Muller Substantiation algorithm –Response Time (ms)**	**Web service translator tool and uppaal tool used for verification of the composed web services-Timed Automata** **(Cambronero et al.** 2011 **; Jin Song et al. ** 2006 **)-Response Time (ms)**	**CWS-PNs methodology for analysis, design and validation of web service choreographies based on** ** (Yang et al. ** 2005 **)** **CPN-Response Time (ms)**
4	5	4	10
8	7	3	20
10	9	5	30
12	12	10	32
14	15	17	43

### 8.4 Performance evaluation-statistical approach

The Reachability problem is solved by the automatic recomposition. When giving the input, it should reach the final state from the starting state. If it does not reach the final state, recomposition will be done and again it will verify the reachability property against the final state. The deadlock property is verified by checking the branching and looping statements in the composed service. If both the properties are verified, then the safety and liveness properties will be automatically verified.

#### 8.4.1 Coefficient of variance

Coefficient of variance is the ratio of standard deviation to the mean.

Table 3 shows the different dead transition values for the different models. ESAM is giving less dead transition value than CPN

**Table 3:** **Dead transition values for different models**

**S. No**	**Number of services**	**Dead transition**	
		**Proposed model using Amend Muller substantiation- hybrid automata**	**Existing model, CWS-PNs methodology used ColoredPetrinet**
1	16	38	63
2	19	37	63
3	34	35	68
4	40	33	78

Calculating Mean, variance and Standard Deviation for Existing and Proposed model using Reachability Factor.

DT for ESAM = Number of states moved – empty string state moved/Total number of states * 100

Or

$$ D{T}_{ama}=\left({F}_s-Esm{}_s\right)/{Q}_0\times 100 $$

**Figure 7:**
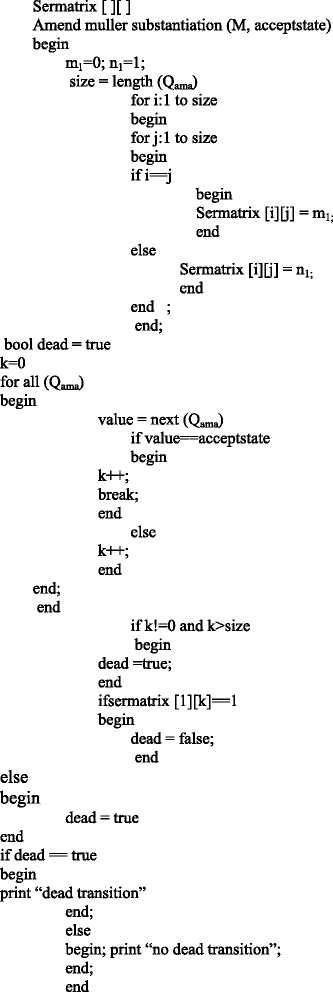
**Dead transition algorithm.**

Let us consider the example 11-5/16*100 = 6/16*100 = 38. Where 6 is the number of states moved using the diagram 5 and 5 is the empty string state is moved referred in the Figure 7, 16 is the total number of states. Finally the output will be the 38%.

Mean $$ \overline{x_{\begin{array}{l}\\ {}\end{array}}}={\displaystyle \sum x/n} $$ = 36. Adding the values of ESAM and sum can be divided by total number of terms ie 4.

Variance ${\sigma}^2={\displaystyle \sum \Big( xi-\overline{x}}\Big){}^2/n$ = 3.75. Subtract each value of ESAM from X_1,_ find the square of the value and finally calculate the sum. This sum can be divided by total number of terms.

Standard Deviation S.D= $\sqrt{\sigma^2}$ = 1.93. Finding square root of the variance is called Standard deviation.

Coefficient of variance CV = Standard Deviation/Mean.

=1.93/36

=0.053

Similarly, calculate the mean, variance and standard deviation for the CPN (Colored Petri Net) Timed Automata) values.

DTesam for Timed Automata = Number of states moved/total number of states * 100

Or

$$ D{T}_{ta}=\left(S{M}_s\right)/{Q}_0\times 100 $$

Let us consider the example 10/16*100 = 63. Where10 is the number of states moved using the diagram 5, divvied by total number of states. Finally the output will be the 63%.

$$ \mathrm{Mean}\ \overline{x}={\displaystyle \sum x/n} = 68 $$

$$ \mathrm{Variance}\ {\sigma}^2={\displaystyle \sum \Big( xi-\overline{x^2}}\Big)/n = 37.5 $$

$$ \mathrm{S}.\mathrm{D}=\sqrt{\sigma^2}=6.12 $$

Coefficient of variance CV = Standard Deviation/Mean.

=6.12/68 = 0.09

Here ESAM value is lesser than the TA value. So TA is riskier thanESAM model

#### 8.4.2 T.Test

It can be used to determine if two sets of data are significantly different from each other, and is most commonly applied when the test statistic would follow a normal distribution if the value of a scaling term in the test statistic were known. When the scaling term is unknown and is replaced by an estimate based on the data, the test statistic (under certain conditions) follows a Student's *t* distribution.

$$ \overline{x_1}={\displaystyle \sum {x}_1/n}=36,\ \mathrm{this}\ \mathrm{value}\ \mathrm{is}\ \mathrm{obtained}\ \mathrm{b}\mathrm{y}\ \mathrm{adding}\ \mathrm{the}\ \mathrm{ESAM}\ \mathrm{value}\mathrm{s}\ \mathrm{and}\ \mathrm{divided}\ \mathrm{b}\mathrm{y}\ \mathrm{number}\ \mathrm{of}\ \mathrm{terms}. $$

$$ {\overline{x}}_2={\displaystyle \sum {x}_2/n}=68,\ \mathrm{similarly}\ \mathrm{f}\mathrm{o}\mathrm{r}\ \mathrm{x}2\ \mathrm{also}.\mathrm{n}1=4\ \mathrm{and}\ \mathrm{n}2=4 $$

$$ {\sigma}^2d={\sigma_1}^2/n1+{\sigma_2}^2/n2, $$

Where

*σ*_1_^2^ is the value of standard deviation for ESAM and *σ*_2_^2^ is the value of standard deviation for TA.

=3.75/4 + 37.5/4

= 10.31

Standard Deviation (S.D) $$ \sigma d=\sqrt{\sigma^2}d=3.21 $$

$$ \mathrm{T}={\overline{x}}_1-\overline{x_2}/\sigma d = 9.96. $$

Where T is obtained by subtracting the value first mean and secondmeans which is divided by standard deviation.

Enter T-table at (n1 + n2-2) degrees of freedom.ie 4 + 4-2 = 6.

The calculated value is 8.33 and Tabulated value for 6 degrees of freedom in p = 0.05 is = 2.45 in table (Foster H).

So, concluding that the calculated value is greater than the tabulated value. So there is a difference between these two..i.e. p = 0.05, 95% difference with the model TA than the ESAM.

## 9 Conclusion and future work

Web services technologies are becoming as popular standard to integrate distributed applications and systems using XML-based standards. Developing applications that support web services interfaces will not be enough to provide complete and coordinated business processes. Thus, we need a new approach to compose these web services together in order to form web services orchestration and processes definition.

Many new standards have been defined to solve this problem, for example BPEL4WS, and WSCI. BPEL4WS provides an initial work for forming an XML specification language for defining and implementing business process workflows for web services. The main problem with most practical approaches to service composition is the verification of (behavioural) correctness of service composition and that too for deterministic system.

In this paper, a new model called ESAM has been proposed and a new algorithm called Amend Stacked Muller Automata Algorithm has been proposed for the verification of web services composition. Web services are created and composed using BPEL4WS, and then it is mapped into Amend Muller Automata notations and transformed into Promela. SPIN tool is used for verifying the composed web services. We experimentally confirmed that our approach can bring 90% reduction of Reach ability, Dead Transitions and verify the safety correctness property effectively in verification process. Hence the proposed model is suitable for a non-deterministic system and also considers the verification part.
